# Common co-morbidities in polymyalgia rheumatica and giant cell arteritis: cross-sectional study in UK Biobank

**DOI:** 10.1093/rap/rkad095

**Published:** 2023-11-02

**Authors:** Charikleia Chatzigeorgiou, John C Taylor, Faye Elliott, Eoin P O’Sullivan, Ann W Morgan, Jennifer H Barrett, Sarah L Mackie

**Affiliations:** School of Medicine, University of Leeds, Leeds, UK; NIHR Leeds Biomedical Research Centre, Leeds Teaching Hospitals NHS Trust, Leeds, UK; School of Medicine, University of Leeds, Leeds, UK; Leeds Institute of Data Analytics, University of Leeds, Leeds, UK; School of Medicine, University of Leeds, Leeds, UK; Department of Ophthalmology, King’s College Hospital NHS Foundation Trust, London, UK; School of Medicine, University of Leeds, Leeds, UK; NIHR Leeds Biomedical Research Centre, Leeds Teaching Hospitals NHS Trust, Leeds, UK; Leeds Institute of Data Analytics, University of Leeds, Leeds, UK; NIHR Leeds Medicines and In Vitro Diagnostics Co-operative, Leeds Teaching Hospitals NHS Trust, Leeds, UK; School of Medicine, University of Leeds, Leeds, UK; NIHR Leeds Biomedical Research Centre, Leeds Teaching Hospitals NHS Trust, Leeds, UK; School of Medicine, University of Leeds, Leeds, UK; NIHR Leeds Biomedical Research Centre, Leeds Teaching Hospitals NHS Trust, Leeds, UK

**Keywords:** epidemiology, polymyalgia rheumatica, giant cell arteritis, co-morbidities, UK Biobank

## Abstract

**Objective:**

The aim was to determine prevalent co-morbidities in cases with PMR or GCA compared with matched controls.

**Methods:**

This was a nested, cross-sectional case–control study within the UK Biobank, which recruited participants aged 40–69 years. Case status was defined as self-reported prior diagnosis of PMR or GCA. Ten controls per case were matched for age, sex, ethnicity and assessment centre. Associations with selected self-reported co-morbidities were studied using conditional logistic regression.

**Results:**

Of PMR (*n* = 1036) or GCA (*n* = 102) cases, 72% were female, 98% White, and 58% reported current use of glucocorticoids. Mean age was 63 years. At the time of the assessment visit, compared with controls, PMR/GCA cases were more likely to report poor general health and at least several days of low mood in the past 2 weeks. PMR was associated with hypothyroidism [odds ratio (OR) = 1.34; 95% CI = 1.07, 1.67] and ever-use of HRT (OR = 1.26; 95% CI = 1.07, 1.47). Regarding common co-morbidities, PMR and GCA were both associated with hypertension (PMR: OR = 1.21; 95% CI = 1.06, 1.39; GCA: OR = 1.86; 95% CI = 1.23, 2.81) and cataract (PMR: OR = 1.51; 95% CI = 1.19, 1.93; GCA: OR = 3.84; 95% CI = 2.23, 6.60). Additionally, GCA was associated with depression (OR = 3.05; 95% CI = 1.59, 5.85). Neither condition was associated with diabetes.

**Conclusion:**

Participants with a history of PMR/GCA, including those not currently taking glucocorticoids, rated their health as poorer than matched controls. Some previously described disease associations (hypothyroidism and early menopause) were replicated. Hypertension and cataract, both of which can be exacerbated by long-term glucocorticoid therapy, were over-represented in both diseases, particularly GCA.

Key messages

- Co-morbidity is common in individuals with self-reported prior diagnosis of PMR/GCA.
- People with PMR/GCA have poorer self-reported health than controls; GCA is associated with depression.
- People with PMR/GCA have more hypertension and cataract than controls.

## Introduction

PMR and GCA are two overlapping, age-associated inflammatory diseases [1]. Both diseases have a peak onset in the mid-seventies and a female predominance [2, 3]. PMR and GCA are treated with long-term oral glucocorticoid therapy [2, 4]. The adverse effects of glucocorticoids include diabetes [5], fracture [4], cardiovascular disease [6] and infection [7]. The co-morbidity profile of patients with PMR/GCA might be determined not only by the effects of the disease and the effects of glucocorticoid treatment, but also by risk (or protective) factors for PMR/GCA.

Hypertension appears to be a risk factor for both PMR [8] and GCA [9]. Conversely, type 2 diabetes has been reported to be protective against both PMR [8] and GCA [10]. Furthermore, the literature on cardiovascular risk after a diagnosis of PMR/GCA appears somewhat contradictory [11–13]. Studies from the UK Clinical Practice Research Datalink (CPRD) have revealed glucocorticoid dose–response relationships for both cardiovascular disease and infection in PMR/GCA [6, 7], but in another CPRD study the overall mortality was not increased in PMR compared with controls [13]. Along similar lines, in a small inception cohort of 359 PMR cases and matched controls, only cataract occurred at greater rates in cases than controls [14]. This evidence has been taken as reassurance that the low-to-medium glucocorticoid doses used for PMR are relatively benign compared with the high doses used for GCA.

Regarding other risk factors, early menopause has been identified as a risk factor for GCA [15], and a history of multiple pregnancies might be protective [16]; it is unclear whether PMR has similar associations.

Current guidelines recommend that GCA should be managed under specialist supervision, whereas PMR is still largely diagnosed and treated in primary care. Mitigating the long-term health and economic impact of GCA and PMR on people and their communities will require a better understanding of the overall needs of patients in both groups.

We sought to explore the multimorbidity burden in people with PMR or GCA, compared with matched controls, in a cross-sectional dataset from the UK Biobank, with a focus on potential GCA/PMR mimics, predisposing factors and glucorticoid-related adverse effects.

## Methods

### Study design and participants

The UK Biobank study is well described elsewhere [17, 18]: 502 649 UK individuals between the ages of 40 and 69 years were recruited to a cohort study (further details can be found in the Supplementary Information (Data source and study population section), available at *Rheumatology Advances in Practice* online). At the baseline assessment visit, all participants completed touchscreen questionnaires, including sociodemographic factors (e.g. age, sex, ethnicity, postcode of residence), lifestyle (e.g. smoking, alcohol) and self-reported medical history. At the time of the data released under this approval, 20 000 of the original 502 649 participants had had a 5-year repeat assessment. Ethical approval for the UK Biobank study was granted by the North West Multi-Centre Research Ethics Committee, the Community Health Index Advisory Group and the Patient Information Advisory Group (ref. 11/NW/0382), and our study was approved by the UK Biobank ethical review committee (application 5237). UK Biobank participants all provided full informed consent.

A nested case–control study was conducted. Cases were defined as individuals reporting a prior diagnosis of PMR or GCA at an assessment visit, regardless of whether they reported current oral glucocorticoid therapy at that visit. For those cases who reported having been diagnosed with PMR or GCA at the 5-year assessment visit, but not at the baseline assessment, only data reported at the 5-year assessment were used in this analysis. For controls, data reported during the baseline assessment were used. Each case was matched by age, sex, assessment centre and ethnicity with 10 controls who did not report PMR or GCA (further details can be found in the Supplementary Fig. S1, available at *Rheumatology Advances in Practice* online).

We tested for associations of GCA or PMR with overall general health (excellent, good, fair or poor), the number of non-cancer illnesses, and self-reported frequency of low mood over the previous 2 weeks (not at all, several days, more than half the days or nearly every day). Based on a literature review of potential GCA/PMR predisposing factors/disease associations, in addition to known glucocorticoid-related adverse effects, we also tested for individual associations with smoking (never/ever), hypothyroidism, average alcohol intake during a typical week over the previous year and BMI. Amongst women, we analysed use of the oral contraceptive pill (ever/never), number of live births (0, 1, 2 or ≥3), history of bilateral oophorectomy, early menopause (<43 years) [15] and use of HRT (ever/never). We also examined associations with the three most prevalent self-reported rheumatic diseases (OA, RA and spondylosis) in PMR/GCA patients.

We tested for associations with self-reported co-morbidities particularly relevant to glucocorticoid therapy, including hypertension, cataract, diabetes, angina, depression, myocardial infarction, stroke, glaucoma, diabetic eye disease and transient ischaemic attack. In the subset of cases with available data on ocular measurements (Supplementary Table S7, available at *Rheumatology Advances in Practice* online), we also compared intraocular pressure and visual acuity.

There are potential inaccuracies in self-reported PMR or GCA status. PMR is well recognized to be mimicked by several other conditions, potentially leading to diagnostic confusion [19, 20]. However, given that co-morbidity is common in the age group affected by PMR [21], the coexistence of a co-morbidity with PMR does not necessarily prevent or invalidate the diagnosis of PMR. We created a list of 21 candidate mimicking conditions (Supplementary Table S1, available at *Rheumatology Advances in Practice* online), selected *a priori* from clinical experience, our prior work [22] and a literature review, and explored their frequency in cases compared with controls. This was used to inform a sensitivity analysis of isolated PMR, in which we excluded the PMR cases with coexistent mimicking conditions that occurred with >1% frequency in the PMR group and showed a significant association with PMR.

### Statistical analysis

Associations were assessed with frequency tables and Pearson’s χ^2^ tests for two independent proportions. The mean and 95% CI were calculated to summarize continuous variables and were compared by Student’s paired *t*-tests. Conditional logistic regression was used to study the association between case–control status and potentially related variables. For categorical variables, the most prevalent category among both cases and controls was chosen as the reference category. Candidate co-morbidities with a frequency of ≥1.0% were analysed further using conditional logistic regression to test for case–control differences (significance level 5%).

For the subset that had detailed ocular measurements at their assessment centre visit, to take account of separate measurements being made for each eye, random-effect models were used to compare intraocular pressure and visual acuity in PMR cases reporting current glucocorticoid use, compared with PMR cases not reporting current glucocorticoid use at the time of the assessment.

Given that the proportion of missing data in the explored variables was ≤5%, no imputation was carried out for missing data [23]. Statistical analysis was performed with Stata v.14.0 statistical software (StataCorp, 2015. *Stata Statistical Software: Release 14*. College Station, TX: StataCorp LP).

## Results

One thousand and thirty-six cases with PMR and 102 cases with GCA, including 23 cases with both conditions, were identified from the self-reported diagnostic codes (Supplementary Fig. S1, available at *Rheumatology Advances in Practice* online). Of these, 72% were female and 98% were classified as White. Assuming a 5% significance level and an exposure prevalence of 20%, these sample sizes give 99% and 31% power, respectively, to detect an odds ratio (OR) of 1.4 in a 1:10 matched sample (Supplementary Tables S2 and S3, available at *Rheumatology Advances in Practice* online).

At the time of the assessment visit, the mean age of PMR/GCA cases was 63 years (median 64 years; interquartile range 61–67 years), and 58% reported currently taking oral glucocorticoid therapy. Forty-one PMR cases (4.0%) and five GCA cases (4.9%) reported taking MTX. Tocilizumab was not approved for GCA at the time of the assessment visits in our dataset.

Co-morbidity was common. The proportion of patients reporting two or more and four or more non-cancer illnesses was, respectively, 66% and 31% of PMR cases (Table 1) and 86% and 46% of those with GCA (Supplementary Table 4, available at *Rheumatology Advances in Practice* online). Cases with PMR (OR = 1.79; 95% CI = 1.49, 2.16; *P* = 8.1 × 10^−10^) or GCA (OR = 2.47; 95% CI = 1.49, 4.09; *P* = 4.5 × 10^−4^) were more likely to have four or more non-cancer illnesses than matched controls (Table 1; Supplementary Table S4, available at *Rheumatology Advances in Practice* online).

**Table 1.** General health of patients with PMR compared with matched controls

	**PMR cases (*n* = 1036) *vs* controls (*n* = 10** **360)**			
	PMR	CTRL	OR (95% CI)	*P*
Self-rating of general health, *n* (%)^a^				
Excellent	44 (4.2)	1612 (15.6)	0.37 (0.27, 0.51)	7.5 × 10^−10^
Good	445 (43.0)	6127 (59.1)	1.00	Reference
Fair	397 (38.3)	2203 (21.3)	2.51 (2.17, 2.90)	<1.0 × 10^−20^
Poor	145 (14.0)	378 (3.6)	5.36 (4.32, 6.67)	<1.0 × 10^−20^
Number of non-cancer illnesses, *n* (%)^a^				
0	145 (14.0)	1966 (19.0)	0.88 (0.71, 1.10)	0.3
1	207 (20.0)	2504 (24.2)	1.00	Reference
2–3	361 (34.8)	3649 (35.2)	1.21 (1.01, 1.45)	0.037
≥4	323 (31.2)	2232 (21.5)	1.79 (1.49, 2.16)	8.1 × 10^−10^
Frequency of depressed mood in the last 2 weeks, *n* (%)^a^				
Not at all	738 (75.0)	7995 (80.8)	1.00	Reference
Several days	193 (19.6)	1534 (15.5)	1.35 (1.14, 1.60)	5.0 × 10^−4^
More than half the days/nearly every day	53 (5.4)	371 (3.7)	1.55 (1.14, 2.09)	0.004

The isolated PMR dataset contains PMR cases who did not report also having GCA, OA or RA, and their matched controls.

a These variables contain <5% missing data; for this analysis, cases were matched with controls by age, sex, ethnicity and assessment centre; the missing category was excluded in calculation of the percentages.

CTRL: controls, matched for age, sex, ethnicity and assessment centre; OR: odds ratio from conditional regression analysis; *P*: *P*-value from the conditional logistic regression.

### Potential PMR/GCA mimics

Initially, we explored the data for co-diagnosis of other conditions that could mimic PMR or GCA. Three of these conditions (OA, RA and spine arthritis/cervical spondylosis) were found to occur with a frequency >1% (Supplementary Table S1, available at *Rheumatology Advances in Practice* online). Of the PMR cases, 38 reported a diagnosis of RA and 168 reported a diagnosis of OA. We found possible associations of each of these potential mimics with both PMR and GCA; as expected, the evidence for an association between RA and PMR was strongest (OR = 2.08; 95% CI = 1.46, 2.98; *P* = 5.1 × 10^−5^), although there was also a statistically significant association between OA and PMR (OR = 1.37; 95% CI = 1.14, 1.63; *P* = 0.001); there was no clear association between spine arthritis and PMR (OR = 1.51; 95% CI = 1.00, 2.27; *P* = 0.05; Table 2). Many of the RA diagnoses came shortly after the PMR diagnosis, although there was a minority of patients who reported being diagnosed with RA substantially before the PMR diagnosis (Supplementary Figs S2 and S3, available at *Rheumatology Advances in Practice* online). Therefore, in subsequent analyses we also conducted sensitivity analyses restricted to isolated PMR (without GCA, OA or RA). The number of GCA cases was too small to perform these sensitivity analyses.

**Table 2.** Associations of PMR and GCA with potential PMR or GCA mimics

Condition [*n* (%)]	PMR dataset				GCA dataset			
	**PMR cases (*n* = 1036) *vs* CTRL (*n* = 10** **360)**				GCA cases (*n* = 102) *vs* CTRL (*n* = 1020)			
	PMR	CTRL	OR (95% CI)	*P*	GCA	CTRL	OR (95% CI)	*P*
OA	168 (16.2)	1293 (12.5)	1.37 (1.14, 1.63)	0.001	19 (18.6)	120 (11.8)	1.73 (1.01, 2.98)	0.046
RA	38 (3.7)	186 (1.8)	2.08 (1.46, 2.98)	5.1 × 10^−5^	<5	12 (1.2)	3.42 (1.08, 10.8)	0.036
Spine arthritis/cervical spondylosis	27 (2.6)	181 (1.75)	1.51 (1.00, 2.27)	0.05	5 (4.9)	14 (1.4)	3.6 (1.30, 10.3)	0.01

CTRL: controls, matched for age, sex, ethnicity and assessment centre; OR: odds ratio from conditional regression analysis; *P*: *P*-value from the conditional logistic regression.

### Candidate predisposing factors

Examining candidate predisposing factors (Table 3), we did not find any association of smoking with PMR or GCA. There was no clear association with frequency of alcohol consumption for PMR, but GCA cases were more likely to report never drinking in the previous year compared with the most common category of once or twice per week (OR = 3.55; 95% CI = 1.82, 6.89; *P* = 1.9 × 10^−4^). We found a significant association between hypothyroidism and PMR (OR = 1.34; 95% CI = 1.07, 1.67; *P* = 0.009), which was not markedly attenuated in the subset with isolated PMR. The number of cases with GCA was too small to confirm or exclude an association with hypothyroidism.

**Table 3.** Candidate PMR and GCA susceptibility factors

	PMR				Isolated PMR (without GCA, RA or OA)				GCA			
	**PMR cases (*n* = 1036) *vs* CTRL (*n* = 10** **360)**				(*n* = 825) *vs* CTRL (*n* = 8250)				GCA cases (*n* = 102) *vs* CTRL (*n* = 1020)			
	PMR	CTRL	OR (95% CI)	*P*	PMR	CTRL	OR (95% CI)	*P*	GCA	CTRL	OR (95% CI)	*P*
Ever smoker, *n* (%)^a^	483 (46.6)	4812 (46.4)	1.01 (0.89, 1.15)	0.8	375 (45.4)	3838 (46.5)	0.96 (0.83, 1.10)	0.6	46 (45.1)	480 (47.1)	0.93 (0.61, 1.40)	0.7
Alcohol consumption, *n* (%)^b^												
Never	116 (11.2)	1035 (10.0)	1.21 (0.95, 1.3)	0.1	86 (10.4)	839 (10.2)	1.06 (0.81, 1.39)	0.7	23 (22.5)	94 (9.2)	3.55 (1.82, 6.89)	1.9 × 10^−4^
Special occasions only	166 (16.0)	1361 (13.1)	1.31 (1.06, 1.62)	0.012	120 (14.5)	1052 (12.8%	1.18 (0.93, 1.50)	0.2	17 (16.7)	142 (13.9)	1.71 (0.86, 3.40)	0.1
One to three times a month	107 (10.3)	1169 (11.3)	0.98 (0.7, 1.24)	0.9	90 (10.9)	923 (11.2)	1.01 (0.78, 1.31)	0.9	13 (12.7)	133 (13.0)	1.34 (0.64, 2.82)	0.4
Once/twice a week	231 (22.3)	2470 (23.8)	1.00	Reference	190 (23.0)	1967 (23.8)	1.00	Reference	19 (18.6)	258 (25.3)	1.00	Reference
Three/four times a week	192 (18.5)	2202 (21.2)	0.93 (0.76, 1.13)	0.5	156 (18.9)	1748 (21.2)	0.92 (0.74, 1.14)	0.5	18 (17.6)	212 (20.8)	1.12 (0.57, 2.20)	0.7
Daily/almost daily	222 (21.4)	2115 (20.4)	1.12 (0.92, 1.36)	0.3	181 (21.9)	1714 (20.8)	1.09 (0.88, 1.35)	0.4	12 (11.8)	180 (17.6)	0.72 (0.41, 1.85)	0.7
Hypothyroidism, *n* (%)	103 (9.9)	796 (7.7)	1.34 (1.07, 1.67)	0.009	84 (10.2)	617 (7.5)	1.42 (1.11, 1.81)	0.005	11 (10.8)	82 (8.0)	1.40 (0.71, 2.76)	0.3

For this analysis, cases were matched with controls by age, sex, ethnicity and assessment centre. The isolated PMR dataset contains PMR cases who did not report also having GCA, OA or RA, and their matched controls.

a Missing data for 6 cases and 44 controls for PMR dataset, 1 case and 6 controls for GCA dataset.

b Missing data for 2 cases and 8 controls for PMR dataset, 1 control in GCA dataset.

CTRL: controls, matched for age, sex, ethnicity and assessment centre; OR: odds ratio from conditional regression analysis; *P*: *P*-value from the conditional logistic regression.

Within females (Table 4), PMR was associated with ever use of HRT, compared with never use (OR = 1.26; 95% CI = 1.07, 1.47; *P* = 0.005), with a similar effect size observed in the isolated PMR sensitivity analysis. A possible association of GCA was seen with early menopause (OR = 1.90; 95% CI = 1.01, 3.59; *P* = 0.047). Neither PMR nor GCA was associated with the other susceptibility factors considered, including number of live births, prior use of oral contraceptive pill or history of bilateral oophorectomy.

**Table 4.** Candidate PMR and GCA susceptibility factors specific to females

	PMR				Isolated PMR (without GCA, RA or OA)				GCA			
	PMR cases (*n* = 747) *vs* CTRL (*n* = 7470)				(*n* = 574) *vs* CTRL (*n* = 5740)				GCA cases (*n* = 73) *vs* CTRL (*n* = 730)			
	PMR	CTRL	OR (95% CI)	*P*	PMR	CTRL	OR (95% CI)	*P*	GCA	CTRL	OR (95% CI)	*P*
Number of live births, *n* (%)^a^												
0	109 (14.6)	1069 (14.3)	1.02 (0.81, 1.29)	0.8	88 (15.3)	853 (14.9)	1.06 (0.82, 1.36)	0.7	14 (19.2)	94 (12.9)	1.55 (0.79, 3.02)	0.2
1	79 (10.6)	865 (11.6)	0.92 (0.71, 1.19)	0.5	68 (11.8)	643 (11.2)	1.08 (0.82, 1.44)	0.6	6 (8.2)	87 (11.9)	0.73 (0.29, 1.83)	0.5
2	339 (45.4)	3412 (45.7)	1.00	Reference	255 (44.4)	2616 (45.6)	1.00	Reference	33 (45.2)	347 (47.5)	1.00	Reference
≥3	219 (29.3)	2119 (28.4)	1.04 (0.87, 1.24)	0.7	162 (28.2)	1625 (28.3)	1.02 (0.83, 1.26)	0.8	20 (27.4)	201 (27.5)	1.05 (0.58-1.90)	0.9
Menopause <43 years, *n* (%)^a^	66 (8.8)	536 (7.2)	1.28 (0.97, 1.70)	0.08	49 (8.5)	405 (7.1)	1.25 (0.91, 1.73)	0.2	16 (21.9)	101 (13.8)	1.90 (1.01, 3.59)	0.047
Ever received menopause hormone therapy, *n* (%)	445 (59.6)	4063 (54.4)	1.26 (1.07, 1.47)	0.005	328 (57.1)	3061 (53.3)	1.19 (0.99, 1.42)	0.06	45 (61.6)	404 (55.3)	1.30 (0.79, 2.15)	0.3
History of bilateral oophorectomy, *n* (%)	87 (11.6)	801 (10.7)	1.11 (0.88, 1.41)	0.4	58 (10.1)	612 (10.7)	0.95 (0.71, 1.26)	0.7	11 (15.1)	75 (10.3)	1.51 (0.76, 3.00)	0.2
Ever taken oral contraceptive pills, *n* (%)	538 (72.0)	5349 (71.6)	1.02 (0.86, 1.22)	0.8	413 (72.0)	4125 (71.9)	1.01 (0.83, 1.23)	0.9	51 (69.9)	524 (71.8)	0.90 (0.52, 1.55)	0.7

For this analysis, cases were matched with controls by age, sex, ethnicity and assessment centre. The isolated PMR dataset contains PMR cases who did not report also having GCA, OA or RA, and their matched controls.

a Variables with this label contain <5% missing data.

CTRL: controls, matched for age, sex, ethnicity and assessment centre; OR: odds ratio from conditional regression analysis; *P*: *P*-value from the conditional logistic regression.

For PMR cases also reporting a diagnosis of hypertension, 67% of cases reported an earlier onset of hypertension than the onset of PMR (Supplementary Fig. S3, available at *Rheumatology Advances in Practice* online). We considered whether hypothyroidism or hypertension might have been identified during the diagnostic work-up for PMR. Although this possibility could not be excluded from the data we had, cases with PMR reported that they were diagnosed with hypothyroidism a mean of ∼10 years before they were diagnosed with PMR (Supplementary Figs S2 and S3, available at *Rheumatology Advances in Practice* online).

### Self-rating of general health and recent low mood

Both PMR (Table 1) and GCA (Supplementary Table S4, available at *Rheumatology Advances in Practice* online) cases were more likely to rate their general health as poor than matched controls. Both PMR (Table 1) and GCA (Supplementary Table S4, available at *Rheumatology Advances in Practice* online) cases were more likely to report low mood for at least several days during the previous 2 weeks. Similar results were observed in sensitivity analysis of isolated PMR cases, and also even when considering only isolated PMR cases no longer taking glucocorticoid therapy (Supplementary Tables S5 and S6, available at *Rheumatology Advances in Practice* online).

### Co-morbidities relevant to glucocorticoid therapy

Both PMR and GCA were significantly associated with hypertension [PMR: OR = 1.21; 95% CI = 1.06, 1.39 (Table 5); GCA: OR = 1.86; 95% CI = 1.23, 2.81 (Table 6)]. In the subset of isolated PMR patients no longer receiving glucocorticoid therapy, similar frequencies of hypertension were observed in cases and in controls (Table 5).

**Table 5.** Co-morbidities relevant to glucocorticoid therapy in PMR, with sensitivity analyses

Co-morbidity	PMR cases *vs* CTRL				Isolated PMR cases *vs* CTRL				Isolated PMR, receiving glucocorticoid treatment *vs* CTRL				Isolated PMR, not receiving glucocorticoid treatment *vs* CTRL			
	PMR (*n* = 1036)	**CTRL (*n* = 10** **360)**	OR (95% CI)	*P*	PMR (*n* = 825)	CTRL (*n* = 8250)	OR (95% CI)	*P*	PMR (*n* = 493)	CTRL (*n* = 4930)	OR (95% CI)	*P*	PMR (*n* = 332)	CTRL (*n* = 3320)	OR (95% CI)	*P*
Hypertension, *n* (%)	380 (36.7)	3354 (32.4)	1.21 (1.06, 1.39)	0.005	287 (34.8)	2683 (32.5)	1.11 (0.95, 1.29)	0.2	184 (37.3)	1614 (32.7)	1.23 (1.01, 1.50)	0.04	103 (31.0)	1069 (32.2)	0.95 (0.74, 1.21)	0.7
Diabetes, *n* (%)	50 (4.8)	526 (5.1)	0.95 (0.70, 1.28)	0.7	41 (5.0)	435 (5.3)	0.94 (0.67, 1.31)	0.7	24 (4.9)	269 (5.5)	0.88 (0.57, 1.36)	0.6	17 (5.1)	166 (5.0)	1.02 (0.61, 1.72)	0.9
Diabetic eye disease, *n* (%)	10 (1.0)	89 (0.9)	1.12 (0.58, 2.17)	0.7	5 (0.6)	82 (1.0)	0.61 (0.25, 1.50)	0.3	<5	46 (0.9)	0.65 (0.20, 2.10)	0.5	<5	36 (1.1)	0.55 (0.13, 2.31)	0.4
Glaucoma, *n* (%)	25 (2.4)	238 (2.3)	1.05 (0.69, 1.60)	0.8	21 (2.5)	182 (2.2)	1.16 (0.73, 1.84)	0.5	15 (3.0)	106 (2.1)	1.43 (0.82, 2.50)	0.2	6 (1.8)	76 (2.3)	0.78 (0.34, 1.82)	0.6
Cataract, *n* (%)	85 (8.2)	583 (5.6)	1.51 (1.19, 1.93)	0.001	64 (7.8)	465 (5.6)	1.42 (1.08, 1.87)	0.01	39 (7.9)	259 (5.2)	1.56 (1.10, 2.23)	0.01	25 (7.5)	206 (6.2)	1.24 (0.80, 1.92)	0.3
Depression, *n* (%)	61 (5.9)	545 (5.3)	1.13 (0.86, 1.48)	0.4	43 (5.2)	444 (5.4)	0.97 (0.70, 1.33)	0.8	20 (4.1)	272 (5.5)	0.72 (0.45, 1.15)	0.2	23 (6.9)	172 (5.2)	1.36 (0.87, 2.14)	0.2
Angina, *n* (%)	60 (5.8)	417 (4.0)	1.42 (1.07, 1.90)	0.016	41 (5.0)	330 (4.0)	1.26 (0.90, 1.77)	0.2	25 (5.1)	204 (4.1)	1.24 (0.81, 1.92)	0.3	16 (4.8)	126 (3.8)	1.29 (0.75, 2.21)	0.3
Transient ischaemic attack, *n* (%)	13 (1.3)	58 (0.6)	2.46 (1.34, 4.52)	0.004	6 (0.7)	37 (0.4)	1.63 (0.68, 3.86)	0.3	<5	20 (0.4)	2.01 (0.68, 5.94)	0.2	<5	17 (0.5)	1.18 (0.27, 5.09)	0.8
Stroke, *n* (%)	21 (2.0)	181 (1.7)	1.11 (0.70, 1.77)	0.6	15 (1.8)	147 (1.8)	1.02 (0.60, 1.74)	0.08	9 (1.8)	85 (1.7)	1.06 (0.53, 2.12)	0.9	6 (1.8)	62 (1.9)	0.97 (0.42, 2.24)	0.9

For this analysis, cases were matched with controls by age, sex, ethnicity and assessment centre.

CTRL: controls, matched for age, sex, ethnicity and assessment centre; OR: odds ratio from conditional regression analysis; *P*: *P*-value from the conditional logistic regression.

**Table 6.** Co-morbidities relevant to glucocorticoid therapy in patients with GCA

Co-morbidity	GCA (*n* = 102	CTRL (*n* = 1020)	OR (95% CI)	*P*
Depression, *n* (%)	13 (12.7)	46 (4.5)	3.05 (1.59, 5.85)	0.001
Hypertension, *n* (%)	50 (49.0)	349 (34.2)	1.86 (1.23, 2.81)	0.003
Diabetes, *n* (%)	10 (9.8)	60 (5.9)	1.75 (0.86, 3.57)	0.1
Diabetic eye disease, *n* (%)	<5	9 (0.88)	4.89 (1.41, 16.97)	0.012
Glaucoma, *n* (%)	5 (4.9)	21 (2.1)	2.45 (0.90, 6.62)	0.08
Cataract, *n* (%)	20 (19.6)	68 (6.7)	3.84 (2.23, 6.60)	9.8 × 10^−6^
Angina, *n* (%)	7 (6.9)	55 (5.4)	1.31 (0.57, 3.01)	0.5
Stroke, *n* (%)	<5	20 (2.0)	2.03 (0.68, 6.04)	0.2
Transient ischaemic attack, *n* (%)	<5	6 (0.6)	7.32 (3.94, 27.5)	0.003
Myocardial infarction, *n* (%)	<5	29 (2.8)	0.68 (0.15, 2.92)	0.6

For this analysis, cases were matched with controls with the same age, sex, ethnic background and place of residence.

CTRL: controls, matched for age, sex, ethnicity and assessment centre; OR: odds ratio from conditional regression analysis; *P*: *P*-value from the conditional logistic regression.

BMI did not differ significantly between PMR/GCA cases and controls (data not shown). Neither PMR nor GCA was significantly associated with diabetes (Tables 5 and 6); GCA was significantly associated with diabetic eye disease (OR = 4.89; 95% CI = 1.41, 16.97), but the frequency of diabetic eye disease was low, giving only very small numbers for the comparison (Table 6).

Both PMR and GCA were significantly associated with cataract [PMR: OR = 1.51; 95% CI = 1.19, 1.93 (Table 5); GCA: OR = 3.84; 95% CI = 2.23, 6.60 (Table 6)]. Both PMR and GCA were also significantly associated with transient ischaemic attack, but the frequency of transient ischaemic attack was low, giving only very small numbers for the comparison (Tables 5 and 6). GCA was significantly associated with depression (OR = 3.05; 95% CI = 1.59, 5.85; *P* = 0.001; Table 6).

### Ocular measurements

No significant difference was seen in ocular measurements comparing cases and controls (data not shown). Comparing the 157 PMR cases currently treated with glucocorticoids and the 108 PMR cases not currently treated with glucocorticoids (Supplementary Table S7, available at *Rheumatology Advances in Practice* online), and excluding PMR cases with concomitant GCA, glucocorticoid treatment was associated with a significantly higher intraocular pressure (*P* = 0.005; intraclass correlation, ρ = 0.66), but the overall magnitude of the difference was of questionable clinical significance.

## Discussion

We studied co-morbidity in UK Biobank participants diagnosed with PMR/GCA in comparison to matched controls. In both diseases, hypertension and cataract were more common than in matched controls, but diabetes was not. Participants with PMR/GCA were more likely to rate their general health as poor and to report low mood for at least several days over the past 2 weeks. Self-report of GCA was associated with self-report of a depression diagnosis. These results underline the importance of treatment, education and support for patients with PMR/GCA. One caveat is that UK Biobank recruited participants aged 40–69 years; therefore, this study is informative about the impact of PMR/GCA on people who are diagnosed relatively young, while still of working age.

One major limitation of this study was the case definition that relied on self-report of GCA or PMR, without information about previous glucocorticoid treatment, which usually forms part of the case definition of PMR in epidemiological studies [2]; in UK Biobank, medication information was available only in relationship to the time of the assessment visit. At the time this study was done, health record linkage data were not available, and therefore validation of self-report diagnosis was not possible. However, UK Biobank had a robust process for ensuring that self-reported diagnoses elicited at interview were mapped to the coding tree only if there was judged to be a very high clinical probability that the diagnosis was correct.

Our data showed an association between PMR and RA; we had expected this from our clinical experience of diagnostic revision owing to overlapping clinical features [22]; shared genetic susceptibility factors have also been proposed [24, 25]. These associations should not be over-interpreted, because PMR and RA are both more likely to be diagnosed in rheumatology clinic attenders, thus introducing a potential collider bias. Nonetheless, we used the observed associations (Table 2) to design sensitivity analyses for isolated PMR, without GCA, RA or OA.

Contrary to previous reports [15, 26], we did not find a clear association with smoking. An association of PMR/GCA with lower alcohol intake was seen, but this might be biased by reverse causation. A previous prospective study had reported that individuals with lower prior alcohol intake were more likely to develop PMR [27]. Future Mendelian randomization studies might help to determine the direction of causality in this relationship.

An association of early menopause with GCA has been previously reported [15], and we replicated this. Various other autoimmune diseases are also associated with early menopause. Furthermore, remission of inflammatory rheumatic diseases can be more difficult to achieve in the perimenopausal state. The pleiotropic effects of female sex hormones are complex to unravel. A previous report [28] had suggested that multiple pregnancies are protective, but we did not observe this.

PMR was associated with prior use of HRT, which is a new finding. Before drawing firm conclusions, further studies in independent datasets are necessary. More detailed analysis of the interval between PMR diagnosis and HRT prescription (dates incomplete in this dataset) might suggest explanations; HRT use as part of an osteoporosis-prevention strategy in glucocorticoid-treated patients, or alternatively, protopathic bias (clinicians initially mistaking PMR symptoms for menopause symptoms). Possible confounding by socio-economic status or ethnicity should also be investigated.

An association between hypothyroidism and PMR has been reported previously [8, 29]. We considered simple detection bias (testing thyroid function in the PMR diagnostic work-up [30]), but in our dataset the two diagnoses usually occurred too far apart in time for this to explain the association. Hypothyroidism shares associations with many autoimmune diseases, including RA [31, 32]. Some authors conceptualize PMR as primarily autoinflammatory [33], but others conceptualize PMR and GCA along a common spectrum [1]. GCA can affect the thyroid artery; further research is needed to determine how often this occurs in patients presenting with PMR.

We did not find a strong association of PMR/GCA with smoking or BMI. Similar to previous reports [8, 34], we found no significant association with diabetes. Hypertension was associated with PMR and GCA, as others had found [8, 35]. The mechanism of the association remains unclear.

Cataract was over-represented in PMR/GCA compared with controls, most probably owing to glucocorticoid therapy. We suggest that patients newly diagnosed with PMR/GCA should have at least a baseline optician check, including intraocular pressure assessment.

Depression was associated with GCA, and self-reported low mood was associated with both PMR and GCA. Other UK studies have shown a high burden of depression in PMR, particularly in those <60 years of age [36], indicating significant unmet need for mental health support.

UK Biobank participants have, in general, higher levels of education and socio-economic status than the general population, which might limit generalizability. UK Biobank recruited participants aged 40–69 years, whereas in population-based studies PMR and GCA occur most commonly in people in their seventies. This feature of the dataset has two consequences. Firstly, our study underestimates the co-morbidity burden of the average patient with PMR/GCA, because age is a risk factor not only for GCA-related blindness [37–40], but also for most glucocorticoid-related adverse effects [41]. Secondly, our study reflects the phenomenon of young PMR; other UK research also showed a higher ratio of PMR to GCA diagnoses in younger patients [27]. Apart from the question of whether this group might be more likely to have undiagnosed, glucocorticoid-masked co-morbidities, including inflammatory polyarthritis [27], younger patients diagnosed with PMR have historically been overlooked in the medical literature; they are likely to have differing health-care needs compared with the average patient with PMR. This is the largest study to date focusing on the health burden of young PMR.

In this study, we explored the multimorbidity burden of UK Biobank participants who self-reported diagnoses of PMR or GCA. There was an excess of hypertension and cataract, but no association with diabetes or BMI. Importantly, mental health impacts were substantial. Many of these effects were seen even in those who were no longer taking glucocorticoids.

This study contributes to the body of knowledge on the multimorbidity burden in PMR/GCA. An important message for clinicians is to pay attention to indices of vascular health and mental health and to consider the potential lasting effects of disease and treatment.

## Supplementary Material

rkad095_Supplementary_Data

## Data Availability

Researchers may apply to UK Biobank to access the data used for this study.

## References

[1] Dejaco C , Duftner C, Buttgereit F, Matteson EL, Dasgupta B. The spectrum of giant cell arteritis and polymyalgia rheumatica: revisiting the concept of the disease. Rheumatology 2017;56:506–15. 27481272 10.1093/rheumatology/kew273

[2] Partington RJ , Muller S, Helliwell T, Mallen CD, Abdul Sultan A. Incidence, prevalence and treatment burden of polymyalgia rheumatica in the UK over two decades: a population-based study. Ann Rheum Dis 2018;77:1750–6. 30297332 10.1136/annrheumdis-2018-213883

[3] Helliwell T , Muller S, Hider SL et al Challenges of diagnosis and management of giant cell arteritis in general practice: a multimethods study. BMJ Open 2018;8:e019320. 10.1136/bmjopen-2017-019320 PMC5829930 29431135

[4] Paskins Z , Whittle R, Sultan AA et al Risk of fracture among patients with polymyalgia rheumatica and giant cell arteritis: a population-based study. BMC Med 2018;16:4. 29316928 10.1186/s12916-017-0987-1 PMC5761155

[5] Wu J , Mackie SL, Pujades-Rodriguez M. Glucocorticoid dose-dependent risk of type 2 diabetes in six immune-mediated inflammatory diseases: a population-based cohort analysis. BMJ Open Diabetes Res Care 2020;8:e001220. 10.1136/bmjdrc-2020-001220 PMC7389515 32719077

[6] Pujades-Rodriguez M , Morgan AW, Cubbon RM, Wu J. Dose-dependent oral glucocorticoid cardiovascular risks in people with immune-mediated inflammatory diseases: a population-based cohort study. PLoS Med 2020;17:e1003432. 33270649 10.1371/journal.pmed.1003432 PMC7714202

[7] Wu J , Keeley A, Mallen C, Morgan AW, Pujades-Rodriguez M. Incidence of infections associated with oral glucocorticoid dose in people diagnosed with polymyalgia rheumatica or giant cell arteritis: a cohort study in England. CMAJ 2019;191:E680–8. 31235489 10.1503/cmaj.190178 PMC6592810

[8] Partington R , Muller S, Mallen CD, Abdul Sultan A, Helliwell T. Comorbidities in patients with polymyalgia rheumatica prior to and following diagnosis: a case control and cohort study. Semin Arthritis Rheum 2020;50:663–72. 32512261 10.1016/j.semarthrit.2020.05.003

[9] Petri H , Nevitt A, Sarsour K, Napalkov P, Collinson N. Incidence of giant cell arteritis and characteristics of patients: data-driven analysis of comorbidities. Arthritis Care Res (Hoboken) 2015;67:390–5. 25132663 10.1002/acr.22429

[10] Wadstrom K , Jacobsson L, Mohammad AJ et al Negative associations for fasting blood glucose, cholesterol and triglyceride levels with the development of giant cell arteritis. Rheumatology 2020;59:3229–36. 32240313 10.1093/rheumatology/keaa080 PMC7590417

[11] Hancock AT , Mallen CD, Muller S et al Risk of vascular events in patients with polymyalgia rheumatica. CMAJ 2014;186:E495–501. 25070989 10.1503/cmaj.140266 PMC4162802

[12] Pujades-Rodriguez M , Duyx B, Thomas SL et al Associations between polymyalgia rheumatica and giant cell arteritis and 12 cardiovascular diseases. Heart 2016;102:383–9. 26786818 10.1136/heartjnl-2015-308514 PMC4789702

[13] Partington R , Muller S, Mallen CD, Abdul Sultan A, Helliwell T. Mortality among patients with polymyalgia rheumatica: a retrospective cohort study. Arthritis Care Res (Hoboken) 2021;73:1853–7. 32741132 10.1002/acr.24403

[14] Shbeeb I , Challah D, Raheel S, Crowson CS, Matteson EL. Comparable rates of glucocorticoid-associated adverse events in patients with polymyalgia rheumatica and comorbidities in the general population. Arthritis Care Res (Hoboken) 2018;70:643–7. 28704600 10.1002/acr.23320 PMC6475501

[15] Larsson K , Mellström D, Nordborg E, Odén A, Nordborg E. Early menopause, low body mass index, and smoking are independent risk factors for developing giant cell arteritis. Ann Rheum Dis 2006;65:529–32. 16126796 10.1136/ard.2005.039404 PMC1798101

[16] Duhaut P , Pinede L, Demolombe-Rague S et al; Groupe de Recherche sur l’Arterite a Cellules Geantes. Giant cell arteritis and cardiovascular risk factors: a multicenter, prospective case-control study. Arthritis Rheum 1998;41:1960–5. 9811050 10.1002/1529-0131(199811)41:11<1960::AID-ART10>3.0.CO;2-X

[17] Sudlow C , Gallacher J, Allen N et al UK biobank: an open access resource for identifying the causes of a wide range of complex diseases of middle and old age. PLoS Med 2015;12:e1001779. 25826379 10.1371/journal.pmed.1001779 PMC4380465

[18] Trehearne A. Genetics, lifestyle and environment. UK Biobank is an open access resource following the lives of 500,000 participants to improve the health of future generations. Bundesgesundheitsblatt Gesundheitsforschung Gesundheitsschutz 2016;59:361–7. 26753864 10.1007/s00103-015-2297-0

[19] Gonzalez-Gay MA , Garcia-Porrua C, Salvarani C, Olivieri I, Hunder GG. The spectrum of conditions mimicking polymyalgia rheumatica in Northwestern Spain. J Rheumatol 2000;27:2179–84. 10990231

[20] Gonzalez-Gay MA , Garcia-Porrua C, Salvarani C, Olivieri I, Hunder GG. Polymyalgia manifestations in different conditions mimicking polymyalgia rheumatica. Clin Exp Rheumatol 2000;18:755–9. 11138344

[21] Piccirillo JF , Vlahiotis A, Barrett LB et al The changing prevalence of comorbidity across the age spectrum. Crit Rev Oncol Hematol 2008;67:124–32. 18375141 10.1016/j.critrevonc.2008.01.013 PMC2536650

[22] Pease CT , Haugeberg G, Morgan AW et al Diagnosing late onset rheumatoid arthritis, polymyalgia rheumatica, and temporal arteritis in patients presenting with polymyalgic symptoms. A prospective longterm evaluation. J Rheumatol 2005;32:1043–6. 15940765

[23] Karahalios A , Baglietto L, Carlin JB, English DR, Simpson JA. A review of the reporting and handling of missing data in cohort studies with repeated assessment of exposure measures. BMC Med Res Methodol 2012;12:96. 22784200 10.1186/1471-2288-12-96 PMC3464662

[24] Weyand CM , Hunder NN, Hicok KC, Hunder GG, Goronzy JJ. HLA–DRB1 alleles in polymyalgia rheumatica, giant cell arteritis, and rheumatoid arthritis. Arthritis Rheum 1994;37:514–20. 8147928 10.1002/art.1780370411

[25] Cutolo M , Cimmino MA, Sulli A. Polymyalgia rheumatica *vs* late-onset rheumatoid arthritis. Rheumatology 2009;48:93–5. 18658202 10.1093/rheumatology/ken294

[26] Brennan DN, Ungprasert P, Warrington KJ, Koster MJ. Smoking as a risk factor for giant cell arteritis: A systematic review and meta-analysis. Semin Arthritis Rheum 2018;48:529–37. 10.1016/j.semarthrit.2018.07.001 30093239

[27] Yates M. The epidemiology of polymyalgia rheumatica and giant cell arteritis. *PhD thesis.* Norwich: University of East Anglia, 2017.

[28] Duhaut P , Pinede L, Demolombe-Rague S et al Cell arteritis and polymyalgia rheumatica: are pregnancies a protective factor? A prospective, multicentre case-control study. GRACG (Groupe de Recherche sur l’Arterite a Cellules Geantes). Rheumatology 1999;38:118–23. 10342623 10.1093/rheumatology/38.2.118

[29] Bowness P , Shotliff K, Middlemiss A, Myles AB. Prevalence of hypothyroidism in patients with polymyalgia rheumatica and giant cell arteritis. Br J Rheumatol 1991;30:349–51. 1913003 10.1093/rheumatology/30.5.349

[30] Dasgupta B , Borg FA, Hassan N, BSR and BHPR Standards, Guidelines and Audit Working Group et al BSR and BHPR guidelines for the management of polymyalgia rheumatica. Rheumatology 2010;49:186–90. 19910443 10.1093/rheumatology/kep303a

[31] Pan XF , Gu JQ, Shan ZY. Increased risk of thyroid autoimmunity in rheumatoid arthritis: a systematic review and meta-analysis. Endocrine 2015;50:79–86. 25645464 10.1007/s12020-015-0533-x

[32] Biro E , Szekanecz Z, Czirjak L et al Association of systemic and thyroid autoimmune diseases. Clin Rheumatol 2006;25:240–5. 16247581 10.1007/s10067-005-1165-y

[33] McGonagle D , McDermott MF. A proposed classification of the immunological diseases. PLoS Med 2006;3:e297. 16942393 10.1371/journal.pmed.0030297 PMC1564298

[34] Ungprasert P , Upala S, Sanguankeo A, Warrington KJ. Patients with giant cell arteritis have a lower prevalence of diabetes mellitus: a systematic review and meta-analysis. Mod Rheumatol 2016;26:410–4. 26381748 10.3109/14397595.2015.1081722

[35] Yates M , Luben R, Hayat S et al Cardiovascular risk factors associated with polymyalgia rheumatica and giant cell arteritis in a prospective cohort: EPIC-Norfolk Study. Rheumatology 2020;59:319–23. 31325308 10.1093/rheumatology/kez289 PMC7243373

[36] Vivekanantham A , Blagojevic-Bucknall M, Clarkson K et al How common is depression in patients with polymyalgia rheumatica? Clin Rheumatol 2018;37:1633–8. 28573368 10.1007/s10067-017-3691-9

[37] Vodopivec I , Rizzo JF 3rd. Ophthalmic manifestations of giant cell arteritis. Rheumatology 2018;57:ii63–72. 29986083 10.1093/rheumatology/kex428

[38] Gonzalez-Gay MA , Miranda-Filloy JA, Lopez-Diaz MJ et al Giant cell arteritis in northwestern Spain: a 25-year epidemiologic study. Medicine (Baltimore) 2007;86:61–8. 17435586 10.1097/md.0b013e31803d1764

[39] Saleh M , Turesson C, Englund M, Merkel PA, Mohammad AJ. Visual complications in patients with biopsy-proven giant cell arteritis: a population-based study. J Rheumatol 2016;43:1559–65. 27252424 10.3899/jrheum.151033 PMC7350139

[40] Chean CS , Prior JA, Helliwell T et al Characteristics of patients with giant cell arteritis who experience visual symptoms. Rheumatol Int 2019;39:1789–96. 31440812 10.1007/s00296-019-04422-5

[41] Duru N , van der Goes MC, Jacobs JW et al EULAR evidence-based and consensus-based recommendations on the management of medium to high-dose glucocorticoid therapy in rheumatic diseases. Ann Rheum Dis 2013;72:1905–13. 23873876 10.1136/annrheumdis-2013-203249

